# Recruitment and retention strategies and the examination of attrition bias in a randomised controlled trial in children’s centres serving families in disadvantaged areas of England

**DOI:** 10.1186/s13063-015-0578-4

**Published:** 2015-03-07

**Authors:** Paul Hindmarch, Adrian Hawkins, Elaine McColl, Mike Hayes, Gosia Majsak-Newman, Joanne Ablewhite, Toity Deave, Denise Kendrick, On behalf of the Keeping Children Safe study group

**Affiliations:** Institute of Health & Society, Baddiley-Clark Building, Newcastle University, Richardson Road, Newcastle upon Tyne, NE2 4AX UK; Child Accident Prevention Trust, Canterbury Court (1.09), 1-3 Brixton Road, London, SW9 6DE UK; Clinical Research & Trials Unit, Norfolk and Norwich University Hospital NHS Foundation Trust, Norwich, NR4 7UY UK; Division of Primary Care, School of Medicine, Floor 13, Tower Building, University Park, Nottingham, NG7 2RD UK; Faculty of Health and Applied Sciences, University of the West of England, Bristol, BS16 1QY UK

**Keywords:** Retention strategies, Attrition bias, RCT, Injury prevention, Pre-school, Children’s centres

## Abstract

**Background:**

Failure to retain participants in randomised controlled trials and longitudinal studies can cause significant methodological problems. We report the recruitment and retention strategies of a randomised controlled trial to promote fire-related injury prevention in families with pre-school children attending children’s centres in disadvantaged areas in England.

**Methods:**

Thirty-six children’s centres were cluster randomised into one of three arms of a 12-month fire-related injury prevention trial. Two arms delivered safety interventions and there was one control arm. Retention rates compared the numbers of participants responding to the 12-month questionnaire to the number recruited to the trial. Multivariable random effects logistic regression was used to explore factors independently associated with participant retention.

**Results:**

The trial exceeded its required sample size through the use of multiple recruitment strategies. All children’s centres remained in the study, despite increased reorganisation. Parent retention was 68% at 12 months, ranging from 65% to 70% across trial arms and from 62% to 74% across trial sites. There was no significant difference in the rates of retention between trial arms (*p* = 0.58) or between trial sites (*p* = 0.16). Retention was significantly lower amongst mothers aged 16–25 years than older mothers [adjusted odds ratio (AOR) 0.57, 95% CI 0.41, 0.78], those living in non-owner occupied accommodation than in owner occupied accommodation (AOR 0.53, 95% CI 0.38, 0.73) and those living in more disadvantaged areas (most versus least disadvantaged quintiles AOR 0.50, 95% CI 0.30, 0.82).

**Conclusions:**

Studies recruiting disadvantaged populations should measure and report attrition by socioeconomic factors to enable determination of the extent of attrition bias and estimation of its potential impact on findings. Where differential attrition is anticipated, consideration should be given to over-sampling during recruitment and targeted and more intensive strategies of participant retention in these sub-groups. In transient populations collection of multiple sources of contact information at recruitment and throughout the study may aid retention.

**Trial registration:**

Clinicaltrials.gov identifier: NCT01452191; Date of registration: 10 October 2011, ISRCTN65067450.

## Background

Failure to retain participants in randomised controlled trials (RCT) and longitudinal studies can cause significant methodological problems, in particular the introduction of attrition bias and loss of statistical power due to the diminution of the achieved sample. Low income and educational levels and lack of health awareness amongst participants have been identified as barriers to retention and require specific strategies [1]. This article reports the recruitment and retention strategies of a cluster RCT with a 1-year follow-up period to promote fire-related injury prevention in families of pre-school children attending children’s centres (CCs) in England.

Systematic reviews have provided guidance for the planning and implementation of effective strategies for participant retention [2-6], all of which recommend using multiple strategies but with slightly different emphases or approaches. Robinson [2] reviewed 21 papers that reported community-based behavioural, medical or drug interventions, or chronic disease conditions, and identified 12 themes from 368 strategies. These themes included monetary incentives, community involvement in the design of the study, minimising participant inconvenience and special tracking methods for follow-up of participants; the authors concluded that use of multiple strategies enhanced retention rates. A Cochrane review [3], assessing methods to increase responses to postal and electronic questionnaires, synthesised evidence from 481 trials evaluating strategies to increase response rates. This is of relevance to our trial as outcome measures were ascertained by use of parent-completed questionnaires. Strategies found to be effective included monetary and non-monetary incentives, shorter questionnaires, pre-notification of the arrival of questionnaire, repeat mailing of questionnaires after non-response, Short Message Service (SMS) reminders, association with a university rather than a government/commercial organisation and assurance of confidentiality. Schoeppe and colleagues [4] reviewed studies on recruitment and retention in community-based behavioural intervention studies [nutrition, tobacco, drug use and human immunodeficiency virus (HIV) prevention] with children aged 3–18 years. They identified effective strategies including: building relationships between researchers and partners who were not part of the research team or participants (e.g. families, children, etc.); minimising the burden on participants; non-study staff acting as project champions and promoting the study in recruitment; optimising follow-up procedures prior to study commencement; incentives; and the design of achievable study protocols within cohesive research teams. Again, the use of multiple strategies was recommended for minimising attrition. Davis *et al.* [5] reviewed 21 studies reporting community-based clinical trials. They also advised using multiple strategies for retention and identified study publicity, incentives and participant tracking as important. They also recommended the need for better reporting of factors affecting participant retention in clinical trials. A more recent Cochrane review [6] reviewed 38 studies addressing retention in RCTs and found that higher monetary incentives (versus lower value incentives) and recorded delivery of questionnaires rather than telephone reminders were successful in enhancing retention in trials using questionnaires to collect outcome data. However, monetary incentives alone, additional questionnaire reminders for participants and priority post over regular post (among others) did not increase retention.

The published literature highlights the importance of high retention rates and that retention can be positively influenced through the use of multiple strategies within studies. While some of the evidence relates to community-based RCTs, there are few reporting findings from trials of injury prevention programmes. This paper reports findings from a cluster RCT involving families with pre-school aged children in disadvantaged areas in England, amongst whom recruitment and retention was anticipated to be challenging.

## Method

This section describes the methods used during the trial and, where appropriate, highlights how these methods meet the 14 recommendations from the literature (See Summary of key retention methods identified from the review of the literature) to optimise participant retention. This is followed by a description of the analysis of factors associated with retention in the trial. Ethical approval for the study was obtained from Nottingham Research Ethics Committee 1, 18/03/11 (study reference no. 09/H0407/14).

### Summary of key retention methods identified from the review of the literature

1. Studies using multiple retention strategies
2. Monetary and non-monetary incentives, notably higher monetary incentives
3. Shorter questionnaires
4. Pre-notifications of the arrival of questionnaire
5. Repeat mailing of questionnaires and recorded delivery of questionnaires rather than telephone reminders
6. SMS reminders
7. Association with a university or other non-government institution
8. Building of relationships between researchers, partners who are not part of the research team and participants
9. Minimising the burden on participants
10. Project champions promoting the study in recruitment
11. Optimising follow-up procedures prior to study commencement
12. The design of feasible study protocols within cohesive research teams
13. Study publicity
14. Participant tracking

### Study aims and design

An injury prevention briefing (IPB) was developed as part of the Keeping Children Safe at Home (KCS) cluster randomised controlled trial by the Centre for Child and Adolescent Health at the University of the West of England in collaboration with the Child Accident Prevention Trust. This IPB provided guidance and exercises for use by children’s centres on the prevention of fire-related injuries in pre-school children. Children’s Centres have a role similar to early years support in other countries (e.g. the Head Start Program in the USA, Canada’s “Early Years Plan” and Head Start in Australia) and the children’s centres that participated in this study were expected to help pre-school children achieve the best start in life through family support, education, health and childcare.

The objective of the trial was to evaluate the effectiveness and cost effectiveness of an educationally based intervention (IPB) with or without facilitation, as a means of changing behaviours to improve fire safety in the home. The primary outcome for the trial was the proportion of families who self-reported, via questionnaire, having a fire escape plan at the 12-month follow-up. A fuller description of the study can be found elsewhere [7].

### Recruitment of children’s centres

The study was carried out in children’s centres (CCs) in study sites in England: Nottingham, Norwich, Newcastle upon Tyne and Bristol. Thirty-six CCs, nine at each study site, were required. Children’s centres set up during the first round of their creation [“first phase” CCs with catchment areas covering the 20% most deprived super output areas (SOAs)] in the four study sites were invited to participate. Where there were insufficient first phase CCs in a study site, the invitation was extended to phase two CCs (those whose catchment areas had more than 50% of children aged under 5 living in one of the 30% most disadvantaged SOAs).

CC managers were sent a letter and information sheet from the lead research site (Nottingham), inviting them to express an interest in taking part in the study. Researchers at each local site followed up expressions of interest with an information-giving session. If a CC was happy to participate, informed consent was obtained.

Randomisation of participating CCs was conducted by the Newcastle Clinical Trials Unit using permutable block randomisation (block size = 9). Each CC was randomised to one of three study arms: IPB plus facilitation (IPB+), IPB only (IPB only) and usual care (control). Randomisation took place after parents were recruited as described below. Sample size calculations indicated that 11 CCs per trial arm (*n* = 33) were required to detect an absolute difference in the percentage of families with a fire escape plan of 20% in either of the two intervention arms compared to the control arm (assuming a control arm prevalence of 42%, as ascertained from a previous study of parents attending CCs in the four areas) [8]. The study had 80% power and a 5% significance level (two-sided), assuming an intraclass correlation coefficient of 0.05 [8] and a cluster size of 20 families per CC. The recruitment of 36 CCs allowed for a potential dropout of one CC per trial arm. To allow for 33% loss to follow-up at 12 months at the family level, the study aimed to recruit 30 families per CC, a total of 1,080 families.

The intervention commenced with the IPB+ arm being provided with a training session on the use of the IPB and its safety exercises. The IPB-only arm was sent the IPB after randomisation but received no training. Those in the control arm continued providing any safety interventions to families as per their normal practice. To ensure that the fire safety messages were given in a ‘real-world’ environment, CC staff carried out all safety interventions provided to parents.

To provide facilitation to implement the IPB and to collect process data, SurveyMonkey™ questionnaires were completed by the IPB+ arm CC study leads, followed by either face-to-face or telephone interviews conducted by research staff at 1, 3 and 8 months post commencement of the intervention. Process data were also collected by SurveyMonkey™ questionnaires at 12-month follow-up in the IPB+ and IPB-only arms.

### Parent recruitment

The recruitment and retention strategies were multifaceted and used approaches previously reported as being effective [2-6], including:

- exploration of barriers and facilitators to implementing health promotion and injury prevention interventions;
- piloting baseline and follow-up questionnaires in CCs;
- provision of small monetary incentives (£5) to families [9] for returned questionnaires;
- ensuring that the study routine was flexible and convenient to study participants (in this case CC staff and families) [9].

Potential parent participants were identified from the databases of all 36 participating CCs. Parents over the age of 16 years who had attended the participating CCs in the previous 3 months, had at least one child under 3 years old (and thus might still be using the CC at the end of the 12-month intervention period) and lived within the catchment area of that CC were eligible to participate. Confirmation that parents were over the age of 16 and that their child was under 3 years of age was obtained from the CC, but data were not collected on characteristics of non-participants as part of the trial.

Study packs (a letter, information sheet, baseline questionnaire and gift voucher claim form) were delivered by post or face to face by a researcher or CC staff, according to local preferences. A postage-paid envelope was provided to return the study documents to the local research team.

Various strategies were used for the initial approach to parents (Table 1), starting with the strategy preferred by the CC. The recruitment rate was frequently monitored, and additional strategies were added if the initial method did not result in the required rate of recruitment within the allowed time frame. In all strategies, parents were encouraged to discuss the study with researchers and ask questions about the study, either by phone or in person, at dedicated sessions at the CC. Informed consent and subsequent completion of the baseline questionnaire occurred in the CC or at parents’ homes with either a researcher or CC staff if a meeting at home had been requested by the family.

**Table 1:** **Recruitment of parents at baseline and follow-up strategies used in questionnaire delivery by trial site**

**Trial site**	**Recruitment strategies used**	**Follow-up strategies used**
**Bristol**	Trial packs posted to parents by researchers. Trial packs given out to parents by researchers in face-to-face sessions in the CC*	Trial packs posted to parents by researchers Trial packs given out to parents by researchers in face-to-face sessions in the CC. Telephone reminders and completion of questionnaires over the phone
**Newcastle**	Trial packs posted to parents by researchers. Trial packs posted to parents by CC staff. Trial packs given out to parents by researchers in face-to-face sessions in the CC	Trial packs posted to parents by researchers. Trial packs given out to parents by researchers in face-to-face sessions in the CC. Telephone reminders and completion of questionnaires over the phone
**Norwich**	Trial packs posted to parents by researchers. Trial packs given out to parents by researchers in face-to-face sessions in the CC. Outreach sessions in parents’ own home by CC staff	Trial packs posted to parents by researchers Trial packs given out by CC staff in the CC Trial packs posted to parents by CC staff Telephone reminders and completion of questionnaires over the phone
**Nottingham**	Trial packs posted to parents by CC staff. Trial packs given out to parents by CC staff in face-to-face sessions in the CC	Trial packs posted to parents by researchers. Trial packs given out to parents by researchers in face-to-face sessions in the CC. Telephone reminders and completion of questionnaires over the phone

*Children’s centre.

Participants were informed that, if they participated, a £5 ‘thank you’ gift voucher for local shops would be given for all returned and completed questionnaires; this was reinforced in the participant information letter. Multiple contact details for parents (address and landline and/or mobile telephone numbers) were collected at baseline to aid follow-up data collection [10-15].

Participants were only considered for recruitment to the trial if they completed and returned both the consent form and the baseline questionnaire.

### Parent baseline and follow-up questionnaires and other materials

Baseline and follow-up questionnaires were piloted in CCs and information collected from parents on the content and how long it took to complete was used to modify the questionnaires [7]. This ensured they were written in a suitable style, easily understandable to the CC clientele [16] and could be completed within a reasonable time frame.

The baseline questionnaire was 16 pages long with 33 individual questions. It asked about economic characteristics, household composition, experience of fire-related accidents, current fire safety behaviours and fire safety equipment, parental knowledge and understanding of what causes fires, and home safety information provided by CCs and parental satisfaction with this information.

The KCS programme ‘branding’ (logo) was used on all envelopes, communications and trial documents to reinforce study identity [5,17].

The study team drew on the relationship between the CC staff and their clientele to identify parents who should not be invited to participate in the trial, for example, where CC staff felt that approaching parents would cause distress or cause an unnecessary burden to the family. Details of why a parent was not approached were not obtained. While recruitment bias was a risk with this strategy, it was felt that to cause an unnecessary burden to a participating family was unacceptable and that to ask CC staff to state reasons for not approaching specific families might breach confidentiality and trust. The utilisation of this type of on-going relationship has previously been shown to increase study participation rates [18].

The 12-month follow-up questionnaire contained the same questions as the baseline questionnaire, except for sociodemographic questions, and in addition contained questions on receipt of safety information on the key messages contained in the IPB, attendance at fire safety sessions, smoking cessation interventions and costs to parents of undertaking the interventions. It comprised 14 pages with 42 questions over four sections. Two reminder 12-month follow-up questionnaires were also developed. The first reminder was a mini questionnaire, comprising six pages, collecting data on fire escape plans, component elements of a fire escape plan, smoke alarm use, and testing and bed time safety routines, while the second reminder was a mini-mini questionnaire, with four pages, collecting data only on fire escape plans and component elements of a fire escape plan.

The follow-up questionnaires were administered using a range of methods, depending on what the CC considered most appropriate for their families and on family preference. In each mailing, a covering letter bearing the study logo and a copy of the study information sheet were enclosed. Study researchers made telephone calls to the families, either sensitising them to the arrival of the questionnaire or reminding them to complete and return it. The pre-notification of questionnaire receipt has been successful previously [10] and also served to remind families of their participation in this study. If families appeared reluctant to respond to the postal request, they were offered the opportunity to complete the mini questionnaire by telephone with a member of the research team. The mini-mini questionnaire was completed over the telephone if the research team felt that the longer questionnaires would not be completed. Persistence in obtaining follow-up data has been reported as a successful strategy in retention of participants at follow-up [19]. If participants did not respond after all three questionnaires had been sent and/or if there was refusal of the offer of telephone completion, this was considered a passive refusal to provide follow-up data.

### Delivery of the intervention by children’s centre staff

The study employed a ‘two-tier’ system of research delivery: CC staff delivered the intervention, while researchers [based in National Health Service (NHS) Trusts and universities in each of the four areas] monitored delivery methods and progress, provided support for delivering the intervention and collected follow-up data. The use of an existing trusted relationship (similar to the one between CC staff and parents) to retain participant contact is a strategy used successfully in previous studies [5] and was considered essential in the delivery of the injury prevention test material. This study used the established relationship between CC staff and their clients to encourage parents to engage with the intervention.

### Sociodemographic patterning of attrition

Sociodemographic patterning of attrition has been noted in previous studies with more disadvantaged participants being more likely to be lost to follow-up [20-22]. To examine whether such biases occurred in this study, we used data on a range of sociodemographic characteristics collected at baseline (see Table 2). Participant postcode data were used to obtain the Index of Multiple Deprivation (IMD) score at the Lower Super Output Area (LSOA) level using the 2010 version [23] with Geoconvert [24] used to match postcodes to LSOAs. If the IMD was not available from this source, it was obtained by entering the postcode into a neighbourhood statistics website [25]. The IMD is a single score for areas that describe an array of measures of social, housing, economic, educational and health deprivation in English neighbourhoods (a high IMD score indicates a high level of deprivation) [26].

**Table 2:** **Univariate and multivariable analysis of baseline factors associated with retention in the trial (row percentages) [missing values]**

**Characteristics [** ***n*** **]**	**Retained** ***n*** **(%)**	**Lost to follow-up** ***n*** **(%)**	**Univariate odds ratio (95% CI)**	**Adjusted odds ratio (95% CI)** **Model with factors significant at** ***p*** **≤ 0.02 on univariate analysis**	**Adjusted odds ratio (95% CI)** **Final model**
Youngest child aged: [25]					
0-1 years	333 (69)	151 (31)	1.00		
1-2 years	405 (67)	198 (33)	0.94 (0.72, 1.23)		
Number of children in family: [41]					
1	383 (71)	159 (29)	1.00		
2	238 (68)	113 (32)	0.88 (0.65, 1.19)	0.93 (0.67, 1.29)	
3	71 (59)	50 (41)	0.59 (0.39, 0.90)	0.68 (0.42, 1.11)	
≥4	37 (65)	20 (35)	0.77 (0.43, 1.40)	0.86 (0.44, 1.68)	
Mother aged: [52]					
Over 25 years	595 (73)	217 (27)	1.00		
16-25 years	131 (53)	117 (47)	0.41 (0.30, 0.56)	0.56 (0.39, 0.81)	0.57 (0.41, 0.78)
Lives in: [17]					
House	616 (70)	265 (30)	1.00		
Flat or other	126 (59)	88 (41)	0.62 (0.45, 0.86)	0.91 (0.62, 1.35)	
Tenure: [25]					
Owner occupied	368 (79)	96 (21)	1.00		
Non-owner occupied	369 (59)	254 (41)	0.39 (0.30, 0.52)	0.65 (0.45, 0.93)	0.53 (0.38, 0.73)
Ethnic group: [50]					
White British	685 (68)	323 (32)	1.00		
Other	32 (59)	22 (41)	0.76 (0.41, 1.40)		
English is first language: [12]					
No	57 (59)	39 (41)	1.00		
Yes	688 (69)	316 (31)	1.49 (0.95, 2.34)	1.42 (0.85, 2.36)	
Single adult household: [43]					
No	622 (71)	255 (29)	1.00		
Yes	109 (57)	83 (43)	0.56 (0.40, 0.78)	0.78 (0.53, 1.13)	
Any smoker in household: [30]					
No	534 (70)	225 (30)	1.00		
Yes	199 (62)	124 (38)	0.72 (0.54, 0.96)	0.90 (0.65, 1.24)	
Household member drinks ≥6 drinks on one occasion: [110]					
No	292 (70)	128 (30)	1.00		
Yes	401 (68)	191 (32)	0.94 (0.71, 1.25)		
IMD quintile: [4]					
1 (2.4-15.6)	176 (79)	46 (21)	1.00		
2 (15.7-25.7)	171 (75)	58 (25)	0.76 (0.48, 1.20)	0.82 (0.49, 1.36)	0.83 (0.51, 1.35)
3 (25.8-34.6)	147 (67)	71 (33)	0.55 (0.35, 0.86)	0.72 (0.43, 1.21)	0.66 (0.41, 1.07)
4 (34.7-46.6)	134 (61)	84 (39)	0.44 (0.28, 0.69)	0.54 (0.33, 0.90)	0.58 (0.35, 0.94)
5 (46.7-74.8)	123 (56)	98 (44)	0.35 (0.23, 0.56)	0.53 (0.31, 0.91)	0.50 (0.30, 0.82)
Had fire escape plan: [19]					
No	436 (69)	196 (31)	0.90 (0.69, 1.17)		
Yes	304 (66)	157 (34)			

### Over-sampling of participants

The over-sampling of participants [10,23], based on assumptions of potential attrition rates, was used to ensure that adequate power was retained for analysis of the primary outcome. The study team did not specifically over-sample those that were thought to be particularly hard to recruit or retain in this study (e.g. more disadvantaged parents); rather, the sample size was inflated to allow for the expected level of attrition across the whole study (33%) [7].

### Study team

Strong professional relationships with CC staff were established by researchers prior to the intervention and developed over the study period. This allowed research staff to maintain close contact in order to monitor study delivery and fidelity to the protocol within each CC.

### The development of the intervention

Several previous studies by the research team were used to inform the development of the intervention. This included a qualitative study interviewing CC staff across the four study sites to explore barriers and facilitators to implementing health promotion and injury prevention interventions. This information from this study helped address barriers and facilitators identified by CC staff and increased researcher understanding of the environment and context in which CC operated, including the parents with which they worked and how these needed to be taken into account in the development of the intervention. Having stakeholders (or their peers) involved in the design of interventions has been reported as giving a sense of ownership to those who deliver it [2,5,11]. The second study interviewed parents attending CCs in the four trial sites to explore fire prevention behaviours and safety procedures used in their homes, to inform choice of the primary outcome measure for the trial and inform the design of the study questionnaires for collecting outcome data [8].

Research staff met with CC managers in the month before randomisation to describe the study and discuss how it fitted with their on-going injury prevention work. The commitment of CC staff was reinforced at these sessions by discussion of how the delivery of the IPB would work in their CCs.

### Developing and maintaining relationships with the children’s centres

The development and maintenance of a good working relationship with trial-associated (but not trial-employed) staff were essential in ensuring the discharge of research duties [14]. Study-specific education of study-associated staff has also been reported as important in engaging parents in studies [19]. While it was essential that trial-associated CC staff were well informed [1], all education and information delivery in this study was designed to minimise the risk of contamination between study arms.

Prior to randomisation, a researcher visited the CC to encourage participation in the trial. A ‘crib sheet’ for these discussions was designed and agreed upon by research staff to ensure consistency in the information given between trial sites. It noted the trial requirements, obligations and benefits to the CC of being part of the research and indicated that safety message delivery could support their usual health promotion activities. The CC staff were made aware of the expectations of participating in the trial, including data collection, delivery and reportage of an IPB-based safety message at a minimum of one session for parents (if they were to be allocated to the IPB+ arm), and that the IPB-only and control arms would need to report all safety sessions delivered to parents.

### Administration of follow-up questionnaires

The 12-month questionnaires were delivered and collected through a mix of face-to-face distribution at trial-specific sessions in the CC, outreach by CC staff and postal delivery (undertaken either by researchers or CC staff) to the participants’ homes depending on the advice of the CC (Table 1). Where face-to-face contact was advocated, some trial sites provided refreshments (cake, biscuits and fruit). Crèche facilities were offered by some CCs to encourage parental attendance. Previous research suggests tangible non-monetary support has been found to be conducive to maintaining participant retention [18]. Where the CC recommended the postal approach, a trial pack containing an initial (full-length) 12-month follow-up questionnaire (with cover letter and pre-paid envelope for questionnaire return) was sent; parents were not required to complete a consent form at follow-up. If no responses were forthcoming, the reminder trial packs were sent and telephone completion offered.

### Trial duties and delivery

It was envisaged that CC staff would have access to and contact with participating parents on a regular basis and would serve as research champions [3] in both the delivery of the intervention and the reporting of trial-specific activity to researchers. All CC contacts were made aware of the trial and its procedures, in general terms, during the original meeting to discuss the study and encourage participation. After randomisation, they were provided with more detailed information appropriate to their trial arms. It was anticipated that the CC staff having on-going contacts with the parents would allow both the planned and opportunistic delivery of fire safety interventions to minimise participant inconvenience [2].

### Incentives for children’s centres

While this article is primarily concerned with the strategies to recruit and retain parents in the trial, the goodwill and continued participation of children’s centres and their staff may also be of interest to trialists designing community-based studies. Children’s centres were offered gift vouchers to the value of £25 at the end of their participation as a gesture of thanks. Those in the IPB+ arm were also provided with contacts for local resources that may have been useful to them in their usual practice (e.g. fire-related local and national contacts, DVDs for use as primary resources and a copy of the “Big Red Book of Accident Prevention” [27]). The IPB-only arm was provided with the IPB and the control arm was also provided with the IPB at the end of follow-up data collection.

### Children’s centre staff follow-up during the intervention period

Follow-up began in the IPB+ arm after the training and after randomisation in the IPB-only and control arms. The CC staff in all trial arms were followed up at specific intervals (by phone) during the course of the year at 1, 3, 8 and 12 months to collect activity logs. The activity logs were used by the CC staff to note the attendance of parent participants at IPB-related safety sessions in both intervention arms and at any safety sessions in the control arm. They also allowed researchers to ascertain the numbers of non-participant parents who received the injury prevention sessions. While these follow-up contacts were primarily to collect activity logs, they also allowed researchers to reinforce the importance of the trial with CC staff.

### Analysis

Response rates are described by trial arm and trial site, with the proportions of parents retained in the trial (defined as receipt of a 12-month follow-up questionnaire) compared using random effect logistic regression models with parents at level 1 and children’s centres at level 2 to allow for clustering at the level of children’s centres. We assessed whether retention varied by trial site within arms by adding an interaction term to the model containing arm and trial site, and assessing its significance using a likelihood ratio test with a *p* value of <0.05 taken as significant. We described response rates by sociodemographic characteristics and by presence of the primary outcome measure at baseline (possession of a fire escape plan). We compared retention in the trial by sociodemographic factors and baseline possession of a fire escape plan using random effects logistic regression with parents at level 1 and children’s centres at level 2 to estimate univariate odds ratios and 95% confidence intervals (CI). The relationship between the IMD score and retention was non-linear, so the IMD score was categorised into quintiles. We assessed the independent effect of factors associated with retention by building a multivariable regression model. All variables with a *p* value of ≤0.2 on univariate analysis were entered into the model and removed in order of least significance. The significance of removing the variable was assessed using a likelihood ratio test with a *p* value of <0.05 taken as significant. Once no more variables could be removed, variables that had been removed were re-entered into the model to assess for significance and retained only if the likelihood ratio test was significant. Models were checked by plotting residual values and sensitivity analyses excluded residuals with absolute values above 2.

## Results

**Figure 1:**
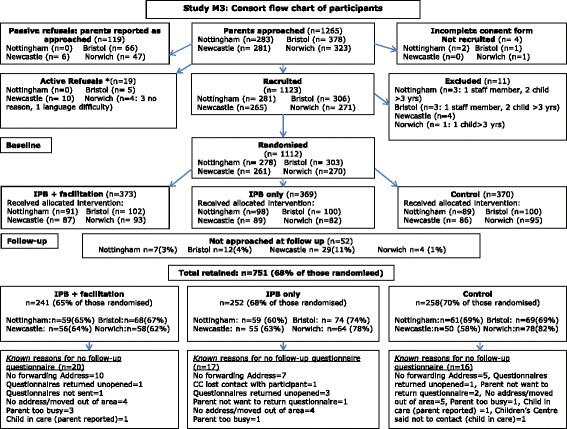
**Flow of parent participants through trial.**

The primary and secondary outcomes from the study have not been reported in this article. They will be reported in the paper presenting the main trial results, which will assess and discuss the potential impact of attrition bias on the trial findings. CC recruitment began in June 2011 and was completed by January 2012. Parent recruitment commenced June 20111 and was completed in May 2012. The CONSORT chart (Figure 1) details the recruitment, randomisation and retention data for the trial with more detailed data on recruitment and retention rates by trial arm and trial site presented in Table 3.

**Table 3:** **Recruitment, retention and attrition rates by study centre and treatment arm**

	**Nott**	**Bris**	**New**	**Nor**	**Total**	**Nott**	**Bris**	**New**	**Nor**	**Total**	**Nott**	**Bris**	**New**	**Nor**	**Total**	**Nott**	**Bris**	**New**	**Nor**	**Total**
	**IPB+**					**IPB only**					**Control**					**Total**				
Baseline*																				
Parents approached	92	128	94	104	**418**	101	135	93	101	**430**	90	115	94	118	**417**	283	378	281	323	**1,265**
Passive refusal	0	20	2	10	**32**	0	34	2	15	**51**	0	12	2	22	**36**	0	66	6	47	**119**
Passive refusal rate	0%	16%	2%	10%	**8%**	0%	25%	2%	15%	**12%**	0%	10%	2%	19%	**9%**	0%	17%	2%	15%	**9%**
Active refusal	0	2	3	1	**6**	0	1	3	2	**6**	0	2	4	1	**7**	0	5	10	4	**19**
Active refusal rate	0%	2%	3%	1%	**1%**	0%	1%	3%	2%	**1%**	0%	2%	4%	1%	**2%**	0%	1%	4%	1%	**2%**
Total refusals	0	22	5	11	**38**	0	35	5	17	**57**	0	14	6	23	**43**	0	71	16	51	**138**
Total refusal rate	0%	17%	5%	11%	**9%**	0%	26%	5%	17%	**13%**	0%	12%	6%	20%	**10%**	0%	19%	6%	16%	**11%**
Incomplete consent form	1	1	0	0	**2**	0	0	0	1	**1**	1	0	0	0	**1**	2	1	0	1	**4**
Incomplete consent rate	1%	1%	0%	0%	**0%**	0%	0%	0%	1%	**0%**	0%	0%	0%	0%	**0%**	1%	0%	0%	0%	**0%**
Excluded/ ineligible	0	2	2	0	**4**	3	0	0	1	**4**	0	1	2	0	**3**	3	3	4	1	**11**
Excluded rate	0%	2%	2%	0%	**1%**	3%	0%	0%	1%	**1%**	0%	0%	2%	0%	**1%**	1%	1%	1%	0%	**1%**
Not recruited	1	25	7	11	**44**	3	35	5	19	**62**	1	15	8	23	**47**	5	75	20	53	**153**
Not recruited rate	1%	20%	8%	11%	**11%**	3%	26%	5%	19%	**14%**	0%	13%	9%	20%	**11%**	2%	20%	7%	16%	**12%**
Participants randomised	91	102	87	93	**373**	98	100	89	82	**369**	89	100	86	95	**370**	278	303	261	270	**1,112**
Recruitment rate	99%	80%	93%	89%	**89%**	97%	74%	96%	81%	**86%**	99%	87%	91%	81%	**89%**	98%	80%	93%	84%	**88%**
12-month follow-up**																				
Parents approached	89	96	75	92	**352**	95	97	78	79	**349**	87	97	80	95	**359**	271	290	233	269	**1,060**
Parents retained	59	68	56	58	**241**	59	74	55	64	**252**	61	69	50	78	**258**	179	211	161	200	**751**
Retention rate (% of those recruited)	65%	67%	64%	62%	**65%**	60%	74%	62%	78%	**68%**	69%	69%	58%	82%	**70%**	64%	70%	62%	74%	**68%**
Not approached	2	6	12	1	**21**	3	3	11	3	**20**	2	3	6	0	**11**	7	12	29	4	**52**
Not approached rate (% of those recruited)	2%	6%	14%	1%	**6%**	3%	3%	12%	4%	**5%**	2%	3%	7%	0%	**3%**	3%	4%	11%	1%	**5%**
Passive refusal	30	24	19	31	**104**	36	22	22	15	**95**	25	26	29	16	**96**	91	72	70	62	**295**
Passive refusal rate	34%	25%	25%	34%	**30%**	38%	23%	28%	19%	**27%**	29%	27%	36%	17%	**27%**	34%	25%	30%	23%	**28%**
Active refusal	0	4	0	2	**6**	0	1	1	0	**2**	1	2	1	1	**5**	1	7	2	3	**13**
Active refusal rate	0%	4%	0%	2%	**2%**	0%	1%	1%	0%	**1%**	1%	2%	1%	1%	**1%**	0%	2%	1%	1%	**1%**
Total refusals	30	28	19	33	**110**	36	23	23	15	**97**	26	28	30	17	**101**	92	79	72	65	**308**
Total refusal rate	34%	29%	25%	36%	**31%**	38%	24%	29%	19%	**28%**	30%	29%	38%	18%	**28%**	34%	27%	31%	24%	**29%**
Questionnaire lost	0	0	0	1	**1**	0	0	0	0	**0**	0	0	0	0	**0**	0	0	0	1	**1**
Total loss to follow-up	32	34	31	35	**132**	39	26	34	18	**117**	28	31	36	17	**112**	99	91	101	70	**361**
Attrition rate (% of those recruited)	35%	33%	36%	38%	**35%**	40%	26%	38%	22%	**32%**	31%	31%	42%	18%	**30%**	36%	30%	39%	26%	**32%**

IPB = Injury prevention briefing, Nott = Nottingham, Bris = Bristol, New = Newcastle, Nor = Norwich.

*The denominator for the rates at baseline is the number of parents approached to participate in the trial.

**The denominator for the rates at follow-up is the number of parents approached except where otherwise stated.

### Recruitment of children’s centres

Eligibility of 100 CCs in England was assessed, four were excluded due to participation in an on-going child safety research project, and 96 were approached by the study team (79 ‘first phase’ and 17 ‘second phase’ CCs). Expressions of interest were received from 57 CCs in the four trial sites [49 ‘first phase’ and 8 ‘second phase’ CCs]. Thirty-six ‘first phase’ CCs and three ‘second phase’ CCs serving disadvantaged areas in Newcastle, Nottingham, Bristol and Norwich were recruited to participate in the trial; we prioritised first phase CCs since these served the most disadvantaged communities. Four CCs in the Nottingham site and two in the Newcastle site shared management structures and operated as single centres, and these centres were therefore allocated in pairs and counted as single centres, giving the total CCs participating as 36. The recruitment of CCs was undertaken over a 3-month period. It is likely that children’s centres that were most interested in and most motivated to prevent injuries were more likely to participate than less interested or motivated centres. We were not able to collect data on injury prevention activity from non-participating children’s centres, so we are unable to know the extent to which this occurred or the possible impact on our findings.

### Recruitment of parents

Parents who passively refused (did not return their questionnaire or consent form or returned a blank questionnaire with no reason given for non-participation) (*n* = 119) at recruitment were reported from all but one site and, where reported, numbered between 6 and 66 per trial site (Figure 1). Active refusals [parents who gave reasons why they did not want to be involved (*n* = 19)] were reported from all but one site and, where reported, numbered between four and ten per trial site. Parents who returned incomplete consent forms (*n* = 4) were excluded from the trial. Known reasons for exclusion from the trial included being a staff member of the CC (*n* = 2) and having a child aged >3 years (*n* = 5). One trial site did not report reasons for exclusion.

In total, 1,112 families were recruited to the trial and randomised. Recruitment rates (expressed as a percentage of those approached) varied between sites: Nottingham 98%; Newcastle 93%; Bristol 80%; Norwich 84%; rates of recruitment by trial arm ranged from 74% to 99%.

### Retention of children’s centres

None of the 36 CCs withdrew from the study. All but one returned all trial questionnaires over the 12-month follow-up period. One did not return a questionnaire at the 8-month contact, citing changes in the organisational structure of the CC and staff pressures. Support and reassurance by trial research staff ensured that the CC agreed to continue with the trial. This CC completed the questionnaire at month 12 and was included in the analysis.

### Retention of parents

Prior to follow-up data collection, the research team contacted all CCs with a list of participating parents to ascertain if any had changed addresses or were no longer appropriate to contact. Across the trial as a whole, 1,060 (95% of participants) were approached for collection of follow-up data. We received 751 completed questionnaires (Table 3) representing 68% of recruited participants. While the majority of responses came from the initial distribution of questionnaires (in person or by post), 20% (*n* = 149) of the total responses came from using the shorter reminders for non-responders (Table 4). Data on reasons for loss to follow-up were not collected systematically, which may be considered a serious limitation. Where these data were recorded (Figure 1), the most frequently cited reasons were lack of up-to-date contact details, parents had moved addresses, the questionnaire was returned as not known at the address, parents being too busy or the child had been taken into care. Reasons for loss to follow-up were similar across trial arms.

**Table 4:** **Returned questionnaires by trial site, arm and questionnaire type**

**Trial centre**	**Trial arm**	**Questionnaire type**				
		**Standard**	**Mini**	**Mini-mini**	**Total by study arm**	**Total by site**
**Bristol**	Control (%)	54 (31)	6 (30)	9 (50)	69 (33)	
	IPB+ (%)	56 (32)	6 (30)	6 (33)	68 (32)	
	IPB only (%)	63 (36)	8 (40)	3 (17)	74 (35)	
	Total (%)	173 (82)	20 (9)	18 (9)		**211 (28)**
**Newcastle**	Control (%)	38 (33)	9 (29)	5 (36)	52 (32)	
	IPB+ (%)	40 (35)	10 (32)	4(29)	54 (34)	
	IPB only (%)	38 (33)	12 (39)	5 (36)	55 (34)	
	Total (%)	116 (72)	31(19)	14 (9)		**161 (21)**
**Norwich**	Control (%)	65 (38)	9 (43)	4 (44)	78 (39)	
	IPB+ (%)	48 (28)	7 (33)	3 (33)	58 (29)	
	IPB only (%)	57 (34)	5 (24)	2 (22)	64 (32)	
	Total (%)	170 (85)	21 (10)	9 (5)		**200 (27)**
**Nottingham**	Control (%)	51 (36)	1 (8)	9 (39)	61 (34)	
	IPB+ (%)	47 (33)	7 (54)	5 (22)	59 (33)	
	IPB only (%)	45 (32)	5 (39)	9 (39)	59 (33)	
	Total (%)	143 (80)	13 (7)	23 (13)		**179 (24)**
**Total by questionnaire type (%)**		602 (80)	85 (11)	64 (9)		**751 (100)**

The 68% retention rate was almost exactly in line with anticipated attrition rates. There was no statistically significant difference in retention rates between trial arms (IPB+ arm = 65%, IPB-only arm = 68%, control arm = 70%; OR comparing IPB+ vs. control 0.79, 95% CI 0.49, 1.27; OR comparing IPB only vs. control 0.96, 95% CI 0.59, 1.55; *p* = 0.58). There was also no statistically significant difference in retention between trial sites (Nottingham = 64%, Bristol = 70%, Newcastle = 62%, Norwich = 74%; *p* = 0.16) and there was no significant interaction between trial site and trial arm (*p* = 0.44).

All trial sites, bar one, used research staff to collect follow-up data in face-to-face sessions at the CC, in addition to other methods for administering the follow-up questionnaires. As there was no significant difference in retention rates between trial sites, no comparison between sites that did and did not use face-to-face sessions can be made. This resource-intensive strategy may not, therefore, be an effective method of increasing retention rates.

### Retention by sociodemographic factors and baseline presence of a fire escape plan

Table 2 shows the relationship between sociodemographic factors and presence of a fire escape plan at baseline and retention, along with unadjusted and adjusted odds ratios. Three factors were significantly independently associated with retention. Families with mothers aged 16–25 years [adjusted odds ratio (AOR): 0.57, 95% CI 0.41, 0.78 compared to families with older mothers], those in non-owner-occupied accommodation (AOR 0.53, 95% CI 0.38, 0.73 compared to those in owner-occupied accommodation) and those living in more disadvantaged areas (AOR comparing most disadvantaged quintile to lease disadvantaged quintile 0.50, 95% CI 0.30, 0.82) were significantly less likely to be retained in the trial.

## Discussion

This study used multiple strategies to optimise recruitment and retention. Research staff used the established relationships between CC leaders and their clientele to recruit, deliver the trial intervention and collect follow-up data. CC staff were able to help with maintaining contact with parents, to advise which parents were not suitable to follow-up and on the best methods of collecting follow-up data. In approaching the CCs to express interest in participating in the study we may have introduced bias; in that only a motivated and interested CC would agree to deliver the study. This may be seen as a limitation.

Reasons for loss to follow-up were not known for most of those not retained within the trial. Non-response due to transience (including relocation out of the CC’s catchment area) and the lack of up-to-date addresses for parents highlight and reinforce the need for a more comprehensive collection of multiple contact points at baseline. The relative inexpensiveness of mobile phones makes changing mobile numbers easy and makes tracing participants more difficult. In this trial, parent participants were not actively encouraged or reminded to notify either research or CC staff of a change of contact details. Although CCs were contacted at 12 months to update the study contacts, some stated that their clientele did not always inform them of changes of address. The sending of Christmas cards to parents also gave them the opportunity to let study team members know of any change in circumstances as well as reminding them of their trial participation. However, no participants were recorded as doing so. Recording email addresses, the contact details of a close friend or family member (who had given informed consent for this purpose) and social media could also be part of a strategy regarding multiple contacts [28]. While SMS messages were used by some CCs to advertise sessions, study arm-specific social media linked directly to the CC could potentially have been used more extensively in our trial. This would have provided a regular and checkable information source to make and maintain participant contact and remind participants of days/dates/times of CC sessions.

### Retention

While there was no difference in the retention rates between sites, it is interesting to note that the two reporting the highest rates (Norwich, 74%; Bristol, 70%) initially had the lowest recruitment rates (84% and 80% respectively). It is possible that CC staff at these sites were more selective in their identification of appropriate participants and that a more ‘committed’ cohort was obtained, facilitating better retention rates. Research staff at these sites also undertook more face-to-face recruitment, and personal contact may also have affected retention. Our study was too small to explore the relationship between recruitment strategies and subsequent retention in any detail but this warrants further investigation in larger studies.

One IPB+ CC in Norwich reported considerable difficulties due to reorganisation and loss of staff, which led to only 43% families recruited to the study being retained. In addition, this CC did not take up the offer of having researchers collect follow-up data at 12 months, potentially placing a larger burden on staff already under pressure at the CC. The CC also reported that many study parents were no longer accessing the CC, so face-to-face data collection was not an option at 12 months. The experience of this CC illustrates previous findings that staff commitment to a study is essential to maximise retention [1].

While it has been reported previously that reminder questionnaires have little effect on increasing responses [6], this strategy is cited by others as positively affecting response rates [2-4]. Our strategy of sending multiple questionnaires accounts for just under 20% of the total responses and should not be underestimated as a tool for optimising retention. Initially our trial estimated attrition of participants at 33%. In over-sampling to accommodate this attrition, the trial ensured that, even with a loss to follow-up of 32% (1% lower than expected), the power of the trial was not compromised.

### Sociodemographic factors associated with retention

A recent systematic review reports on 26 studies and 6 reviews on factors associated with attrition in research studies amongst socioeconomically disadvantaged groups [29]. The review highlights the barriers to retention of such participants in research studies. Our finding of a lower retention rate amongst young mothers, those living in non-owner-occupied accommodation and in more disadvantaged areas is consistent with the findings from this review and with several previous studies that have also reported lower retention rates amongst younger than older mothers [1,30]. The review reported that the greatest challenge for researchers was in maintaining contact with study participants, whose lives often had a transient nature with frequent changes of address and telephone numbers. Other common barriers included difficulties with transport, lack of child care, problems with taking time off work for study participation or research requirements competing and losing out to the priorities of daily life [29]. Previous reviews suggest that injuries and exposures that increase the risk of injuries are both more common with social disadvantage [31,32]. Hence our finding that more disadvantaged parents were less likely to be retained in our study may mean our estimates of prevalence of fire safety practices at follow-up may overestimate the prevalence amongst trial participants. However, we found no significant difference in retention rates by trial arm, suggesting the differential retention rates by social group should not affect our estimates of the effectiveness of the intervention.

## Conclusion

Using a range of recruitment strategies enabled our trial to exceed its sample size requirements despite recruiting in socioeconomically disadvantaged areas. This was helpful as 32% of recruited participants were lost to follow-up. Attrition did not differ between treatment arms, but there was evidence of social patterning of attrition, with the more disadvantaged being less likely to be retained in the trial. Studies recruiting disadvantaged populations should measure and report on attrition by socioeconomic variables to enable the extent of attrition bias and the potential impact on results to be assessed. Where differential attrition is anticipated from participants in more disadvantaged areas, consideration should be given to differential over-sampling at baseline to allow for greater loss from this subset of the study sample and/or to targeted and more intensive methods of participant retention in these sub-groups. This study showed that no single strategy could be identified that, in isolation, optimised recruitment and retention; we conclude that a multifaceted approach should be considered when undertaking trials of this kind.

## Abbreviations

AOR: Adjusted odds ratio
CC: Children's centres
CI: Confidence interval
DVD: Digital versatile disc
HIV: Human immunodeficiency virus
IMD: Indices of multiple deprivation
IPB: Injury prevention briefing
KCS: Keeping children safe
LSOA: Lower super output areas
NHS: National Health Service
NIHR: National Institute of Health Research
RCT: Randomised controlled trial
SMS: Short message service
SOA: Super output areas
